# A proteomic study of the effect of UV-B on the regulatory mechanism of flavonoids metabolism in pea seedlings

**DOI:** 10.3389/fnut.2023.1184732

**Published:** 2023-05-15

**Authors:** Xin Fu, Yinghao Xu, Ming Lu

**Affiliations:** ^1^Food and Processing Research Institute, Liaoning Academy of Agricultural Sciences, Shenyang, China; ^2^College of Food, Shenyang Agricultural University, Shenyang, China

**Keywords:** proteomics, seedlings, phytohormones, flavonoids, UV-B, metabolic pathways

## Abstract

This study aimed to investigate the mechanism of response of pea seedlings to UV-B stress from a proteomic perspective. In this experiment, we measured the growth of pea seedlings in two groups affected by UV-B and unaffected by UV-B and conducted proteomic analysis. The results showed that the ascorbic acid content of UV-B-irradiated pea seedlings increased by 19.0%; the relative content of flavonoids increased by 112.4%; the length of edible parts decreased by 14.2%, and the elongation of roots increased by 11.4%. Proteomics studies showed a significant increase in the levels of CHI, F3'5'H, F3H, F3'H, C4H, and CHR, which are key enzymes for flavonoid synthesis. RT-qPCR indicated that the expression of the regulatory genes of these enzymes was significantly upregulated. This study provided a basis for further studies on the flavonoid response mechanism in pea seedlings during UV stress.

## 1. Introduction

Pea (*Pisum sativum L*.) is an annual herb of the legume family. The main components of pea are starch, fiber, and proteins (1), with a protein content of up to 30% or more (average 22.3%) (2). Therefore, pea is a high-quality legume with high nutrient content and is widely consumed because it is cheap and easily available. Currently, it is legume crop with the fourth largest cultivation area globally. Peas are rich and high in phenolic substances, especially in dark seeded varieties (3, 4), and the main phenolic components of peas also include flavonoid compounds (5). Humans have mastered the production of hydroponic bean sprouts since more than 2000 years ago and have a long history of consuming bean sprouts (6). To some extent, sprouting can change the nutritional structure of beans and improve the bioavailability of vitamins and minerals (7). Most of the nutrients in beans exhibit an upward trend and often a significant increase during the germination process (8). Pea seedlings, obtained by germination of pea seeds, are a long-standing traditional vegetable food in Asian countries such as China. Pea seedlings are easy to prepare and grow and are inexpensive. The content of phenolic compounds, particularly flavonoids, is better in sprouted peas than in unsprouted ones (9).

Ultraviolet-B (UV-B) has a wavelength of 290–320 nm, accounting for 1.5% of the total energy of solar radiation, and is often used to process plant-based foods. UV-B radiation has the strongest biological effects. It causes various physiological and ecological changes in various plants (10), which in most cases are harmful (11). In response to the UV-B-induced damage in plants, plant-related secondary metabolism is accordingly changed. Studies have reported that UV-absorbing compounds such as polyphenols and flavonoids are the main target products of plants in response to UV stress. As reactive oxygen scavengers in plants, flavonoids are mainly distributed in leaves and play an important role in resistance of plants to UV stress (12). Additionally, flavonoids in leaves can accumulate in plants as phytochemicals (13) and have a protective effect against microbial damage (14).

Flavonoids are a class of monomers that play a defensive role in plants (15). Studies have reported that flavonoids are mainly distributed in tissues and organs that are sensitive to UV radiation, such as the epidermal cell layer of the plant body near the stem and leaves, and are the main UV-resistance substances in plants (16). The main role of flavonoids is to absorb harmful UV radiation and protect the photosynthetic organs of plants to maintain the normal photosynthesis in plants (17). In plant foods, phenylalanine enters a variety of different flavonoid synthesis pathways after the formation of chalcone by the phenylalanate pathway (18), forming a rich variety of flavonoids. The effect of UV-B on flavonoid synthesis is reflected at the transcriptional level, a process in which UV-B can activate photoreceptors to generate signals, and light signal transducers can be kinases (19), nitrogen oxides (20) or phosphodiesterases, which can activate the Chalcone synthase (CHS) promoter and promote the expression of CHS genes (21). In addition, UV-B also elevates flavonoid 3'-hydroxylase (F3'H) activity, which subsequently affects the transcription of cinnamic acid 4-hydroxylase (C4H) and chalcone isomerase (CHI), of which C4H may be a key step in the flavonoid biosynthetic pathway (22).

Proteomics is an emerging technique for the systematic, high-throughput, large-scale study of the composition and function of all proteins in a particular type of cell or tissue. Proteins are the carriers of all life activities. To some extent, protein levels can reflect the state of the organism, metabolism, and response to stimuli. Although protein translation is regulated by gene expression, transcript levels do not represent the level of cellular active proteins (23). Moreover, the transcript levels do not reflect the post-translational modification process of proteins, which plays a key role in protein activity and function (24). In the life activities of plants, proteins are the most direct functional executors responsible for implementing various specific functions. Due to the spatial and temporal specificity of protein expression, transcript and protein levels may not necessarily coincide. Therefore, while studying the effects of UV radiation on the metabolic processes of related substances, which cannot be studied simply at the gene level.

In recent years, many studies have reported the use of UV irradiation on processed food. However, the effect of UV-B on pea seedlings is relatively unexplored. Particularly, no systematic study has been conducted to assess the mechanism of response of pea seedlings UV light from a proteomic perspective. This study provided new insights into this, and the data obtained can help to understand the mechanisms regulating the response of plants to UV radiation. In this study, we examined the physiological and biochemical parameters of pea seedlings before and after UV-B treatment, performed proteomic analysis to identify the differential proteins to understand the regulatory mechanisms associated with the response of pea seedlings to UV-B, and studied the expression of regulatory genes to determine the effects of UV-B on the metabolic pathways of related substances.

## 2. Materials and methods

### 2.1. Material handling

A kind of pea of variety Liao Pea No. 31 (G0000314) from Liaoning province of China was used in this study. The materials were full-blown, free of lesions and scars, and similar in size and shape. The experimental materials were evenly divided into two groups: ultraviolet treatment group (UV) and control group (CK). The materials were sterilized by using a 0.1% potassium permanganate solution soaked for 20 min, and incubated at a constant temperature of 25°C with no light before germination. After growing to 2 cm, pea seedlings were incubated under light from a plant growth incubator with a light source consisting of a mixture of 1,000 lx of red, green and blue colors. At this point, daily irradiation with UV light of wavelength 253.7 nm was started for 10 min/day for the UV group, while the CK group is left untreated. The water was changed once every 24 h and sprayed with warm water at 25°C once every 12 h. The samples were frozen in liquid nitrogen and stored at −80°C. Day 0 was set as the day before the UV group received its first UV irradiation after germination.

### 2.2. Inspection of length and weight of pea seedlings

Sampling was done at a fixed time each day. Overall, 50 pea seedlings were randomly selected from the germination tray, and the length of root and edible part and weight of edible part were measured and averaged. The length of edible part refers to the height of the plant from the base of the stem to the highest point of natural growth, and root length refers to the distance between the seed emergence and the farthest point of the main root. Weight of edible part refers to the weight of the seedling portion of the plant from the base of the stem.

### 2.3. Ascorbic acid content

Ascorbic acid content was determined using a vitamin C assay kit (Suzhou Grace Biotechnology Co., Ltd., China).

### 2.4. Flavonoids content

The extraction and determination of flavonoids were based on the method of Cao et al. (25) with some modifications. In total, 6.25 g of pea seedlings were accurately weighed into a mortar. To this, precooled (at 4°C) 1% concentrated HCl in methanol (HCl-methanol solution) was added and ground on an ice bath. The material was placed in a 500-mL volumetric flask; the mortar was washed with 1% HCl-methanol solution and the contents were placed in the same volumetric flask. Make up the volume of the mixture with 1% hydrochloric acid-methanol solution until it reaches the scale of 500 mL, and then mix well. The mixture was shaken four times and allowed to stand at 4°C for 20 min; further, the mixture was filtered and filtrate was collected. The absorbance of the solution was measured using a UV spectrophotometer with 1% HCl-methanol solution as the blank reference, and the absorbance of the solution was measured at 325 nm. The absorbance value indicated the relative content of flavonoids, which was compared among the samples.

### 2.5. Proteomics analysis

The proteome was explored using the Tandem mass tag (TMT) labeling quantitative assay. Briefly the steps were as follows. The proteomics analysis process was referred to the literature (26).

#### 2.5.1. Protein extraction

Proteins in the samples were extracted and quantified using Pierce™ BCA Protein Assay Kit (ThermoFisher Scientific, USA). The BCA kit was used to prepare BCA working solution and standard protein solutions with different mass concentrations of 0, 0.125, 0.250, 0.500, 0.750, 1.000, 1.500, and 2.000 mg/mL. 2 μL of each sample was taken and mixed with 18 μL of H_2_O, and 200 μL of BCA working solution was added. Mix with shaking, react at 37°C for 30 min, and read the absorbance at 562 nm.

#### 2.5.2. Enzymatic alkylation and labeling

Add Bond-Breaker™ TCEP Solution (TCEP) reductant (ThermoFisher Scientific, USA) at a final concentration of 10 mmol/L and react for 60 min at 37°C. Add iodine acetamide (Sigma, Germany) at a final concentration of 40 mmol/L and react for 40 min at room temperature, protected from light acetamide (Sigma, Germany) at room temperature for 40 min. Add pre-chilled acetone (acetone: sample volume = 6:1) to each tube and precipitate for 4 h at −20°C. Centrifuge the precipitate for 20 min at 10,000 × *g* using a Centrifuge 5430R (Eppendorf, Germany). The samples were fully dissolved with 50 mmol/L Triethylammonium bicarbonate buffer (TEAB, Sigma, Germany), and Trypsin was added at a mass ratio of 1:50 (enzyme: protein) and digested overnight at 37°C. The samples were labeled with TMT and mixed, and the TMT reagent was removed at −20°C (ThermoFisher Scientific, USA) to room temperature, add acetonitrile (ThermoFisher Scientific, USA), vortex centrifuge and add one tube of TMT reagent per 100 μg of peptide (TMT10-126 labeling, 127N labeling A2, 127C labeling A3, and 128N labeling B1). Incubate for 2 h at room temperature; add hydroxylamine, react for 15 min at room temperature, mix equal amounts of labeled products in one tube and evacuate using LNG-T88 vacuum concentrator (Taicang Huamei Biochemical Instrument Factory, China).

#### 2.5.3. Reversed-phase liquid chromatography (RPLC) one-dimensional separation

The peptide samples were re-solubilized with UPLC loading buffer and separated in high pH liquid phase using ACQUITY UPLC BEH C18 Column (Waters, USA). Two percent acetonitrile (ammonia to pH 10) for phase A, 80% acetonitrile (ammonia to pH 10) for phase B, 0–2 min, 100% A; 2–17 min, 0–3.8% B; 17–35 min, 3.8–24% B; 35–38 min, 24–30% B; 38–39 min, 30–43% B; 39–40 min, 43–100% B; 40–46 min, 100%-0 B. The UV detection wavelength was 214 nm, the volume flow rate was 200 μL/min, and the elution time was 66 min. A total of 28 fractions were collected according to peak shape and time, combined into 14 fractions, and concentrated by vacuum centrifugation.

#### 2.5.4. Liquid phase tandem mass spectrometry (LC/MS)

The second dimension was analyzed using an Evosep One liquid chromatograph (Evosep, Denmark) in tandem with an Orbitrap Exploris 480 mass spectrometer (Thermo, USA). Peptides were solubilized with mass spectrometry loading buffer, and then separated by a C18 column (150 μm × 15 cm, Evosep, Denmark) for 120 min at a volume flow rate of 300 μL/min. The liquid phase gradient eluted with 2% acetonitrile (plus 0.1% formic acid) in phase A and 80% acetonitrile (plus 0.1% formic acid) in phase B for 0–1 min, 0–5% B; 1–63 min, 5–23% B; 63–88 min, 23–48% B; 88–89 min, 48–100% B; 89–95 min, 100% B. The MS and MS/MS acquisitions were automatically switched between MS and MS/MS with mass spectral resolutions of 70 and 35 K, respectively. MS was performed with a full sweep (m/z 350–1,300), and the parent ion was selected top20 for secondary fragmentation, with a dynamic exclusion time of 18 s.

#### 2.5.5. Protein search results and bioinformatic analysis

The raw files from the mass spectrometry downlink were analyzed by ProteomeDiscoverer™ Software 2.4. The false discovery rate (FDR) for peptide identification during library search is set to FDR ≤ 0.01. The protein contains at least one specific peptide. Trans-Proteomic Pipeline (TPP) was used to filter and card the results obtained from the database search to improve the correctness of protein identification and sequencing using mass spectrometry. The obtained mass spectrometric data were analyzed using Xcalibur™Software3.0 Qual Browser, and fold change (FC) and *p*-values were selected as reference standards for the screening of differential proteins between groups. The criteria for differences were FC > 1.2 or < 0.83 and *p* < 0.05. Functional annotation analysis was performed using BLAST2GO software. All proteins and protein sequences obtained using mass spectrometry were compared with seven databases (Uniprot, NR, GO, KEGG, COG, Pfam, and String) and subcellular-localization-related databases to obtain annotation information of the proteins in each database. Enrichment analysis was performed on the proteins in the protein set using the software Goatools, using the Fisher exact test. The Benjamini and Hochberg (BH) method was used to correct *p*-values, and significant enrichment was considered when the corrected *p*-value was < 0.05.

### 2.6. RT-qPCR to detect the transcripts of genes regulating differential proteins

To examine the transcripts of genes regulating differential proteins and to investigate the process by which differences occur in gene expression, we selected proteins that were significantly different between groups and were the key enzymes for flavonoid metabolism. Further, quantitative reverse transcription PCR (RT-qPCR) was performed using the genes regulating these proteins.

EF-1a was selected as an internal reference gene. The transcript levels were calculated using the 2–ΔΔCT method. In the first step, the RNA from the samples was isolated and reverse transcribed to cDNA using the reagent HiScript Q RT SuperMix for qPCR (+gDNA wiper). In the second step, RT-qPCR was performed using ChamQ SYBR Color qPCR Master Mix (2 ×) in a total volume of 20 μL, containing 10 μL 2 × ChamQ SYBR Color qPCR Master Mix, 2 μL cDNA template, 0.4 μL 50 × ROX Reference Dye 2, 0.8 μL primer F (5 μM), 0.8 μL primer R (5 μM), and 6 μL ddH_2_O. Overall, 40 cycles were performed in a fluorescent qPCR instrument at 95°C for 5s, 55°C for 30s, and 72°C for 40s. The primers used are given in Table 1.

**Table 1:** Primer profile.

**Gene**	**Primer sequences**	**Length**
Psat2g003880.1	F: 5′ GATGTGGCTGATCTATTGCC 3′	287
	R: 5′ TCCATAACCTCATTTGCTCC 3′	
Psat6g238360.1	F: 5′ ACATCATTTCAGGTCCATTT 3′	239
	R: 5′ TAACCCTAGTATTCCATCAG 3′	
Psat2g003920	F: 5′ AGTCAATAAGATAGCGATGC 3′	185
	R: 5′ TTGTTGTTTAGAGGGTAGGT 3′	
Psat4g099800	F: 5′ TCCCACCTACCTTATCCTAC 3′	151
	R: 5′ GGAGCGGAGTGACAGTGTTG 3′	
Psat7g077640.1	F: 5′ GCATACGAGGTTGGATTGCC 3′	156
	R: 5′CATTGCAACTGAACCAGCCCACAAG 3′	
Psat5g201640.1	F: 5′ CCAGGTGGTGACAAGTATGA 3′	138
	R: 5′ CACAGATAATGGCACGGCTC 3′	
Psat6g237840	F: 5′ ATTTCCGCTTGGTCTTTATT 3′	161
	R: 5′ CTTGAGTCGATGATAGGTGC 3′	
Psat2g050720.1	F: 5′ GTATTACTATCGGTCGTCTGG 3′	283
	R: 5′CTTGGCTTAGCAACAATGGTGGAAT 3′	
Psat5g069280.1	F: 5′ GTCATCACATATCATTTCCCTG 3′	107
	R: 5′ TCAACAACATTGGCATTCTC 3′	
EF-1a	F: 5′ GATTGACAGGCGATCTGGTAAGG 3′	-
	R: 5′ GGCTGCTGCTTTGGTGACCTT 3′	

### 2.7. Data analysis

Three biological replicates and three technical replicates were used for each sample. Data processing was performed using Excel 2010. Significance was determined using SPSS Statistics 26, and figures were drawn using OriginPro 9.5.

## 3. Results and discussion

### 3.1. Physiological and biochemical indices of pea seedlings under UV-B irradiation

**Figure 1:**
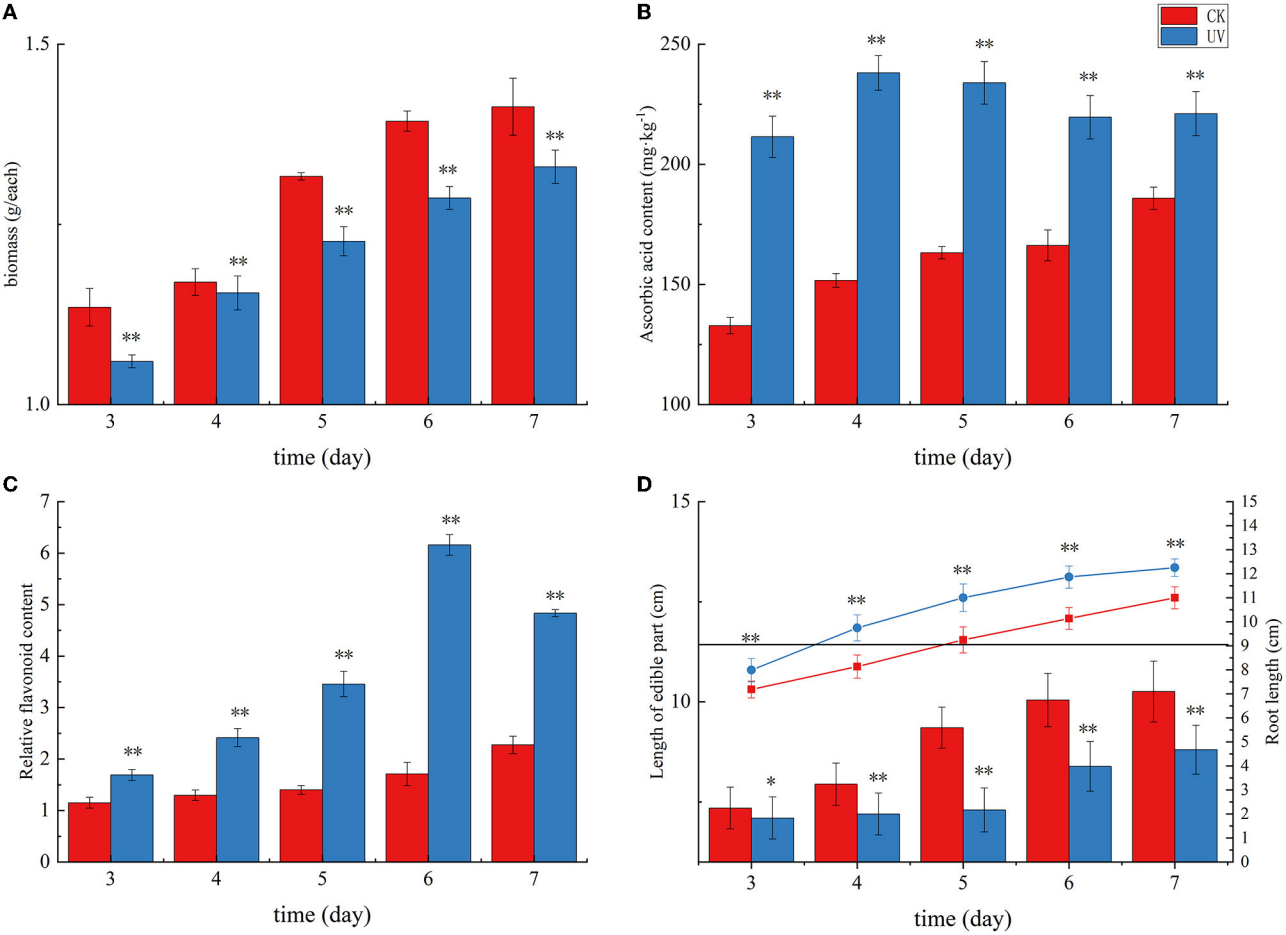
The growth status and yield of pea seedlings under UVB irradiation. Changes in weight **(A)**, ascorbic acid content **(B)**, flavonoid content **(C)**, and length of edible part and root **(D)** of UV-B-irradiated pea seedlings. * indicates significant difference (*p* < 0.05), ** indicates highly significant difference (*p* < 0.01).

The pea germination process is part of the nutritional growth stage of the plant, and the change in weight reflects the combined effect of photosynthetic capacity and physiological, biochemical, and growth factors. UV irradiation is essentially an abiotic stress that can affect the secondary metabolism of plants, and subtle effects on the morphology and weight production of the plants accumulate, causing significant changes in weight. As shown in Figure 1A, the weight of pea seedlings under UV irradiation was significantly lower than that of the control, indicating that UV irradiation had a significant negative effect on the weight of pea seedlings (*p* < 0.01), which is consistent with previous studies on rice (27). In this study, the average weight of the CK group was 1.41 g at day 7, whereas the average weight of the UV group was 1.33 g. The weight of pea seedlings after UV-B treatment decreased by 5.7%. Under the influence of UV-B, the total weight was significantly reduced due to UV-B radiation, which may be caused by changes in morphological and physiological processes caused by UV-B triggering adaptive protective mechanisms in plants (28). The reduction in biomass accumulation in UV-B-treated plants is due to, among others, the reduction in leaf area. Whereas, this phenomenon is not always detrimental (29), in addition, the reduction in weight may also be related to the inhibition of photosynthesis (30), in which the effects of UV-B on pea seedlings accumulate and eventually cause significant changes in weight.

As shown in Figure 1, plants exposed to UV radiation exhibited an increase in the levels of stress-resistant substances, including ascorbic acid and flavonoids, in response to this abiotic stress. Ascorbic acid in plants mainly plays a role in protecting photosynthesis and improving plant resistance to adversity. Its content reflects the environmental stress faced by plants. The ascorbic acid content in the CK group exhibited a gradual increase with germination time, and that in the UV group exhibited an overall increasing trend and then decreasing trend with increasing germination time (Figure 1B). The ascorbic acid content in the UV-irradiated pea seedlings was significantly higher than that in the control (*p* < 0.01). This may be due to the fact that UV-B is involved in the gene expression of systems such as ascorbic acid-glutathione (31), and its irradiation induces changes in the activity of a series of enzymes in the ascorbic acid metabolic pathway, and their combined effect results in an elevated ascorbic acid content, which is consistent with the previous studies on strawberries (32) and tomato fruits (33). The UV-B treatment enhanced the ascorbic acid content in pea seedlings by 19.0%, reaching 221.14 mg/kg. Plant flavonoids are secondary metabolites synthesized by the plant itself. These compounds play a role as antioxidants in plants and in plants' response to abiotic stresses. The effect of UV irradiation on the flavonoid content in pea seedlings is shown in Figure 1C. Compare with the CK group, the relative content of flavonoids in the UV group significantly increased (*p* < 0.01). One of the responses of plants to UV-B damage mechanism is to increase the level of flavonoids in plants (34), probably because UV-B activates the relevant genes and enzymes in the flavonoid pathway, which leads to increased synthesis of flavonoids, and the increased content of flavonoids can reduce the net flux of UV-B radiation into the body, and also can scavenge more excess free radicals in the body. The relative flavonoid content in UV-B-treated pea seedlings increased by 112.4%, there was a substantial increase, which is consistent with similar studies in most plants, such as postharvest citrus (35), grapes (36), and Arabidopsis (37).

The effect of UV irradiation on the length of root and edible parts in pea seedlings at various times is shown in Figure 1D. The plant height of pea seedlings exhibited a significant decrease under UV-B irradiation (*p* < 0.01). UV-B irradiation alters phytochrome activity and subsequent phytohormone synthesis (38), and phytohormones have the potential to affect plant root length along with aboveground plant height. In our study, the length of edible part of pea seedlings decreased by 14.2% after UV-B treatment, which is consistent with the studies on wheat (39) and pepper crop (40). A significant increase (by 11.4%) in root length was observed after UV-B treatment (*p* < 0.01), which is consistent with previous studies (41).

### 3.2. Comprehensive analysis of the proteome

**Figure 2:**
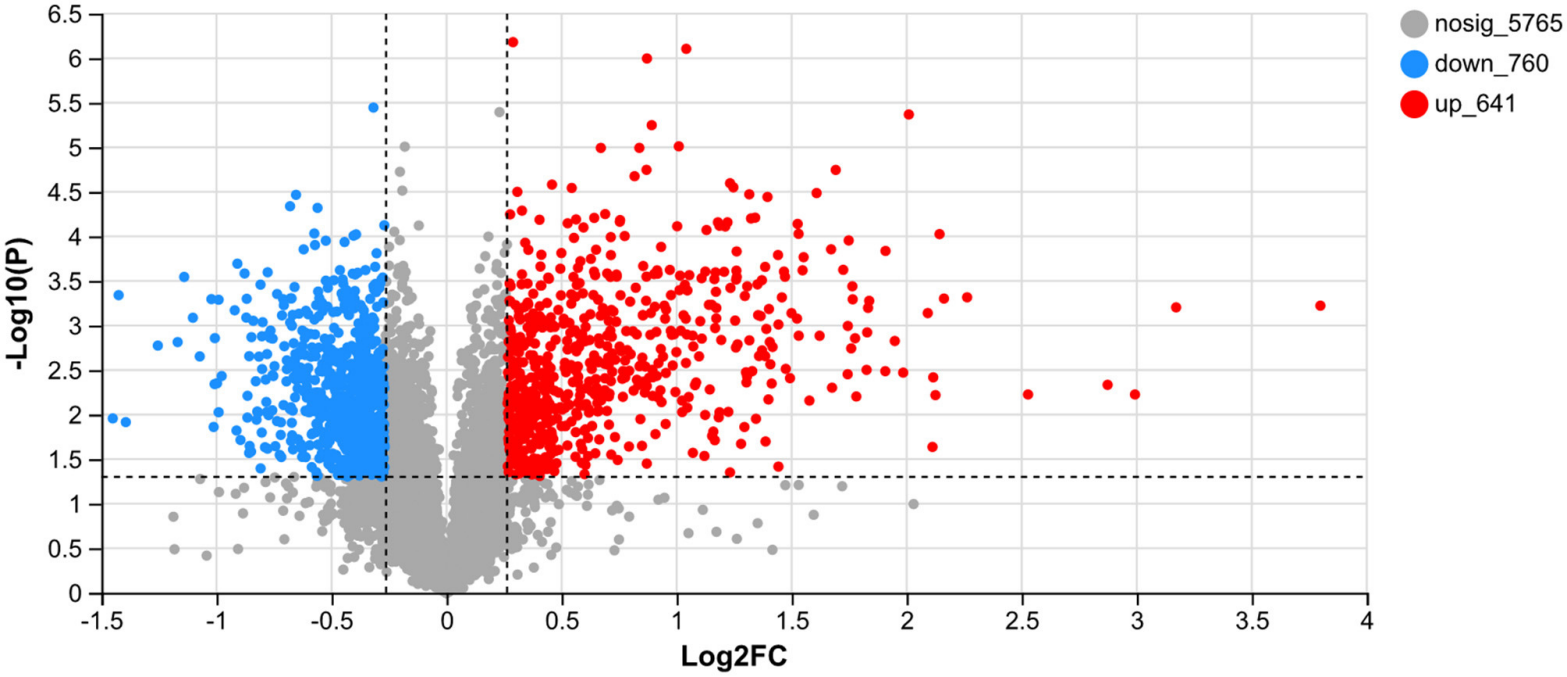
Differentially expressed proteins between the CK and UV groups.

UV-B exhibits a significant biological effect. The differential proteins between the CK and UV groups were assessed (Figure 2). A total of 7,166 proteins were detected in the samples, of which, 1,401 proteins exhibited significantly different expression (641 upregulated and 760 downregulated). A large number of proteins in the UV group exhibited substantial differences under UV-B irradiation compared with the CK group, which implies that UV-B has a significant effect on the growth of pea seedlings (*p* < 0.05). This is consistent with a study on more than 200 plants (42).

**Figure 3:**
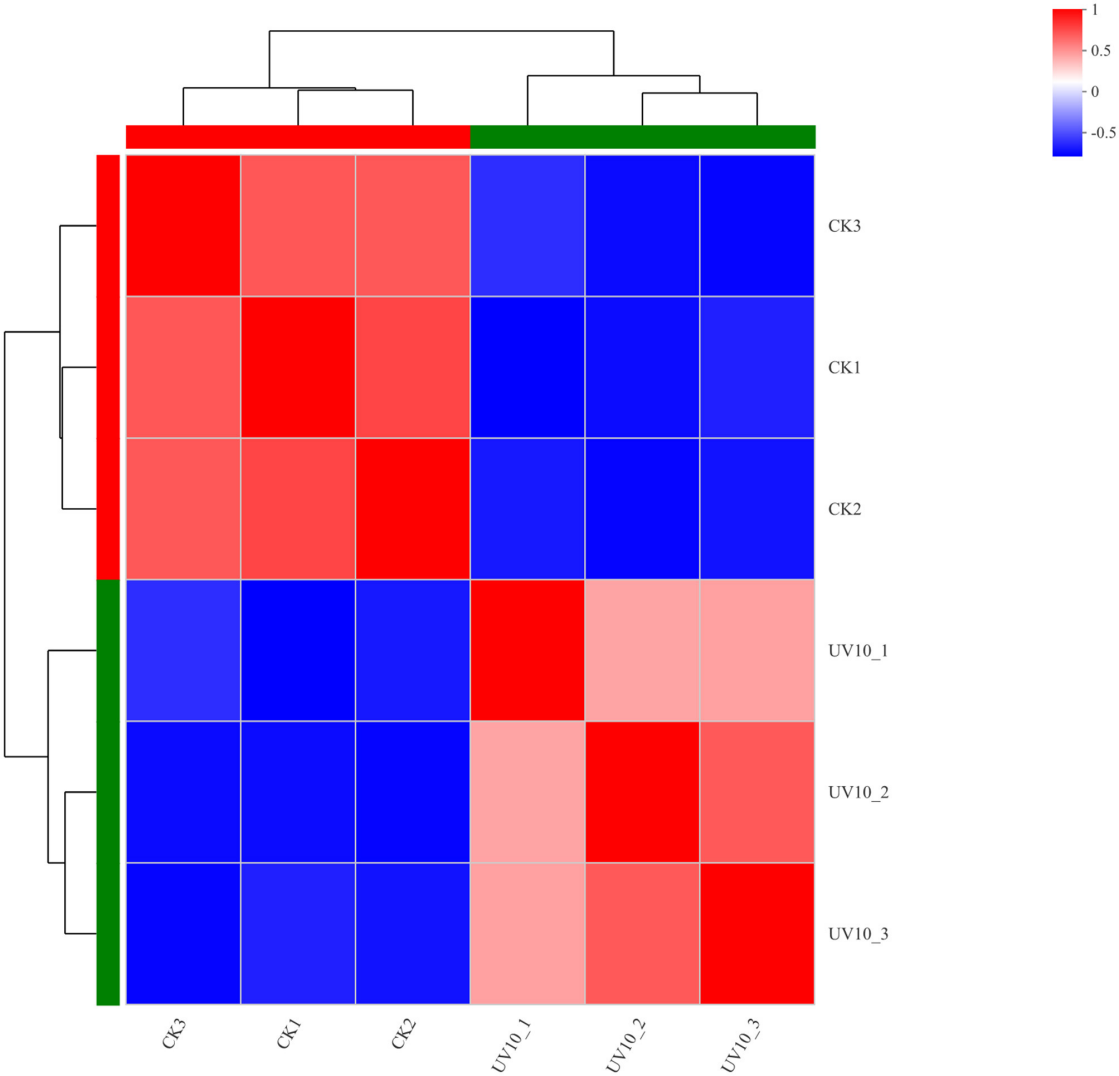
Correlation analysis results between the CK and UV groups.

To verify that the variation among biological replicates is as expected by the experimental design and to provide a basic reference for differential protein analysis, expression correlation analysis was performed on the proteomic data to quantitatively analyze the degree of variation in protein composition among samples (Figure 3), with various shades of color representing correlations. The proteins in the CK and UV groups exhibited significant difference in expression (*p* < 0.05). The correlated protein composition within the CK and UV groups was highly similar, which indicated that the proteome was of high quality and met the conditions for subsequent analysis.

### 3.3. Functional annotation analysis

**Figure 4:**
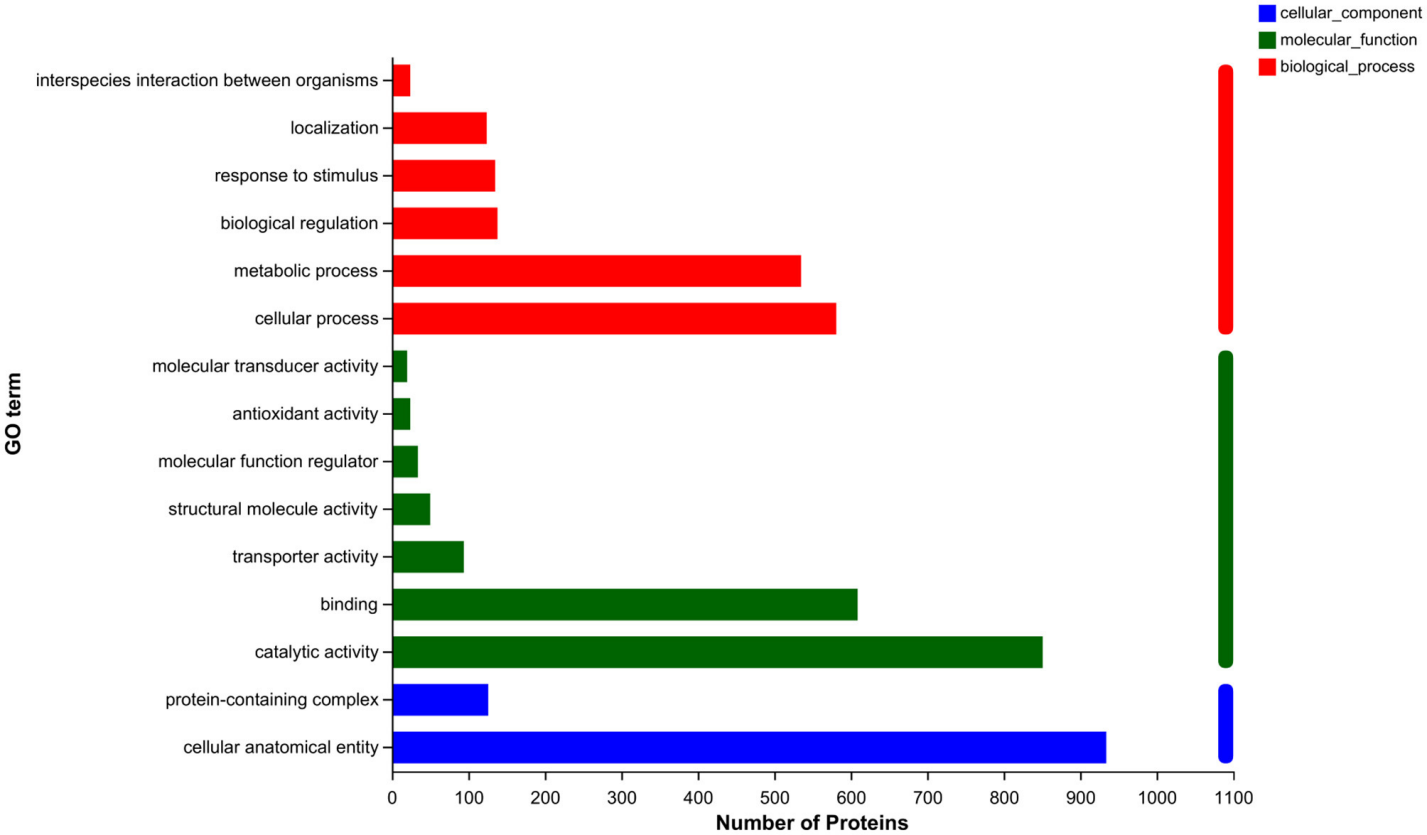
GO annotation analysis.

To elucidate the metabolic pathways in which the differential proteins were involved and the functions performed by these proteins, the proteins were identified based on mass spectrometry and all the proteins obtained were compared with the GO database. A simple comparison was made with the transcriptomic data generated from related studies. As shown in Figure 4, the top 15 GO-annotated functional groups in the proteome in terms of abundance were counted. They were interspecies interaction between organisms (24), localization (124), response to stimulus (135), biological regulation (138), metabolic process (535), cellular process (581), molecular transducer activity (20), antioxidant activity (24), molecular function regulation (34), structural molecule activity (50), transporter activity (94), binding (609), catalytic activity (851), protein-containing complex (126), and cellular anatomical entity (934).

### 3.4. Functional enrichment analysis

**Figure 5:**
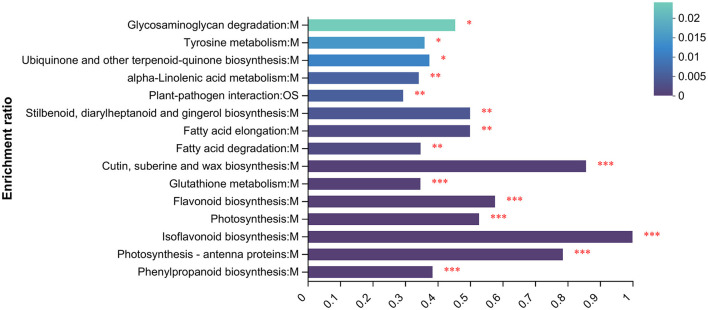
KEGG enrichment analysis. *p < 0.05, **p < 0.01, ***p < 0.001.

To understand the functions of proteins under UV irradiation, enrichment analysis of the proteome was performed using the KEGG database (Figure 5). The top 15 KEGG-enriched metabolic pathways with the highest abundance in the proteome were calculated. They were glycosaminoglycan degradation, tyrosine metabolism, ubiquinone and other terpenoid-quinone biosynthesis, alpha-linolenic acid metabolism, plant–pathogen interaction, stilbenaid, diaryiheptanoid and gingerol biosynthesis, fatty acid elongationfatty acid degradation, cutin, suberine and wax biosynthesis, glutathione metabolism, flavonoid biosynthesis, photosynthesis, isoflavonoid biosynthesis, photasynthesis-antenna proteins, and phenylpropanoid biosynthesis.

### 3.5. Identification and analysis of differential proteins

#### 3.5.1. Differential proteins involved in ascorbic acid metabolism

Table 2 presented the differential proteins involved in ascorbic acid metabolism under UV irradiation. Significant differences (*p* < 0.05) were shown in the levels of six proteins involved in ascorbic acid metabolism under UV-B irradiation. Among them, there were two key enzymes on the metabolic pathway, for GDP-mannose-3'5' isomerase (GME) and inositol oxygenase (MIOX). Among them, GME is involved in the L-galactose pathway, the most important pathway for ascorbic acid synthesis (43), and its levels are positively correlated with ascorbic acid levels (44). In the present study, UV-B irradiation significantly changed (*p* < 0.05) the level of GME, which was increased in the cytoplasm and decreased outside the cell. The possible reason is that more ascorbic acid is needed intracellularly to cope with UV-B damage, thus raising the amount of synthetase, or it is possible that extracellular enzymes have entered the cell. As for MIOX, it is involved in the inositol pathway of the ascorbic acid synthesis pathway (45). In the presence of MIOX, inositol generates glucuronide to participate in the synthesis of ascorbic acid, and in the present study, UV-B irradiation increased the level of MIOX enzyme, whose subcellular localization was in the cytoplasm.

**Table 2:** Differential proteins involved in ascorbic acid metabolism.

**Accession ID**	**FC (UV/CK)**	**Regulation**	**Description and location**
Psat5g086480.1	2.394089	Up	Aldehyde dehydrogenase family 3 member H1 (cytoplasmic)
Psat7g019040.10	2.0115	Up	Inositol oxygenase 2 (cytoplasmic)
Psat6g146120.1	1.539806	Up	GDP-mannose-3,5-epimerase (cytoplasmic)
Psat2g125080.1	0.747047	Down	GDP-mannose 3,5-epimerase (extracellular)
Psat4g126400.1	0.819389	Down	Aldehyde dehydrogenase family 2 member B4 (mitochondrial)
Psat4g097080.1	0.498334	Down	Aldehyde dehydrogenase family 3 member F1 isoform X1 (cytoplasmic)

#### 3.5.2. Differential proteins involved in plant hormone signaling

The differential proteins involved in plant hormone signaling under UV irradiation were assessed (Table 3). Compared with the effect of UV-B on transcriptome, only a small fraction of phytohormone proteins, mainly the corresponding protein kinases, response proteins, and transporter proteins, exhibited differences in protein levels after UV-B treatment. Among them, the level of AUX1 was significantly downregulated (*p* < 0.05) in the Golgi after UV-B irradiation, perhaps the response to UV-B stress makes the synthesis of this substance compromised (46). AUX1 is a high-affinity growth hormone transporter protein (47) that plays an important role in growth hormone transport (48), and the deficiency of AUX1 may inhibit the synergistic transport of growth hormone and affect plant growth and development. Moreover, UV-B irradiation significantly (*p* < 0.05) and substantially upregulated SAUR72 localized in mitochondria, which has not been reported in other plant-related studies. SAUR72 is mainly present during hypocotyl and young root development (49), and studies on Arabidopsis have reported that overexpressed SAUR72 promotes primary root elongation (50), and this may also be one of the reasons for the elongation of the plant roots.

**Table 3:** Differential proteins involved in plant hormone signaling.

**Accession ID**	**FC (UV/CK)**	**Regulation**	**Description and location**
Psat1g006320.1	8.269977	Up	Auxin-responsive protein SAUR72 (mitochondrial)
Psat6g169520.1	1.218075	Up	Histidine-containing phosphotransfer protein 1 isoform X1 (cytoplasmic)
Psat7g033560.1	0.733105	Down	Serine/threonine-protein kinase BSK7 (cytoplasmic)
Psat1g186240.1	0.745518	Down	Putative His-Asp phosphotransfer protein (cytoplasmic)
Psat3g130520.1	0.576531	Down	AUX1-like auxin influx carrier protein (Golgi apparatus)
Psat6g078720.1	0.770962	Down	Serine/threonine-protein kinase CTR1 isoform X1 (cytoplasmic)

#### 3.5.3. Differential proteins involved in flavonoid metabolism

The most obvious feature of the plant response to UV stress is the increased accumulation of reactive oxygen species, which act as oxygen radicals in living organisms and which can cause severe damage to plant proteins, membranes, DNA and other cellular components (51). In the present study, the differential proteins involved in the synthesis of flavonoids under UV irradiation were assessed (Table 4). A total of 14 proteins involved in the synthesis of flavonoids exhibited differences in their levels after UV irradiation. The enzymes involved in the biosynthesis of flavonoid compounds, particularly some key enzymes, exhibited a significant increase in their levels. The possible reason is that the damaging effect of UV-B irradiation on DNA may induce the accumulation of UV-absorbing flavonoid compounds and other phenolic compounds. UV-B irradiation seems to induce the accumulation of flavonoid compounds due to the activation of phenylpropanoids. Flavonoid accumulation is attributed to the activation of the phenylpropanoid pathway to tolerate UV harm (52). Among them, the levels of CHI and flavonoid 3-hydroxylase (F3H) were significantly increased (*p* < 0.05), which is consistent with subcellular localization in the cytoplasm. The level of C4H was significantly increased (*p* < 0.05), which is consistent with its subcellular localization in the endoplasmic reticulum. Moreover, the levels of flavonoid 3',5'-hydroxylase (F3'5'H), F3'H, and chalcone reductase (CHR) were significantly increased in the cytoplasm (*p* < 0.05), plasma membrane, and peroxisome, respectively, which is not reported in related studies of other plants. Furthermore, it is interesting to note that the levels of shikimate O-hydroxycinnamoyltransferase were significantly increased in the peroxisomes (*p* < 0.05) but significantly decreased in the cytoplasm (*p* < 0.05).

**Table 4:** Differential proteins involved in flavonoid synthesis.

**Accession ID**	**FC (UV/CK)**	**Regulation**	**Description and location**
Psat2g003880.1^*^	14.591291	Up	Chalcone reductase (peroxisomal) CHR
Psat6g238360.1^*^	3.289113	Up	Chalcone–flavonone isomerase 1 (cytoplasmic) CHI 1
Psat2g003920.1^*^	3.501469	Up	Chalcone reductase (peroxisomal) CHR
Psat4g099800.1^*^	2.993007	Up	Flavonol-3-hydroxylase (cytoplasmic) F3H
Psat7g077640.1^*^	2.881323	Up	Flavonoid 3',5'-hydroxylase (cytoplasmic) F3'5'H
Psat1g049080.1	2.607458	Up	Quercetin 3'-O-methyltransferase (cytoplasmic)
Psat5g201640.1^*^	1.6716	Up	Flavonoid 3'-hydroxylase (plasma membrane) CYP75B1-F3'H
Psat3g088280.1	1.577715	Up	Shikimate O-hydroxycinnamoyltransferase (peroxisomal)
Psat7g080040.1	1.781155	Up	Hypothetical protein TSUD_68100 (cytoplasmic)
Psat5g069280.1^*^	1.627053	Up	Probable chalcone–flavonone isomerase 3 (cytoplasmic) CHI 3
Psat2g050720.1^*^	1.350324	Up	Cinnamic acid 4-hydroxylase (endoplasmic reticulum) CYP73A-C4H
Psat6g237840.1^*^	1.236944	Up	Chalcone–flavonone isomerase 2 (cytoplasmic) CHI 2
Psat5g308360.1	0.683429	Down	Shikimate O-hydroxycinnamoyltransferase (cytoplasmic)
Psat5g183720.1	0.631683	Down	Shikimate O-hydroxycinnamoyltransferase (cytoplasmic)

^*^This protein is a key enzyme in the metabolic pathway.

### 3.6. RT-qPCR validation of regulatory gene expression of differential proteins

**Figure 6:**
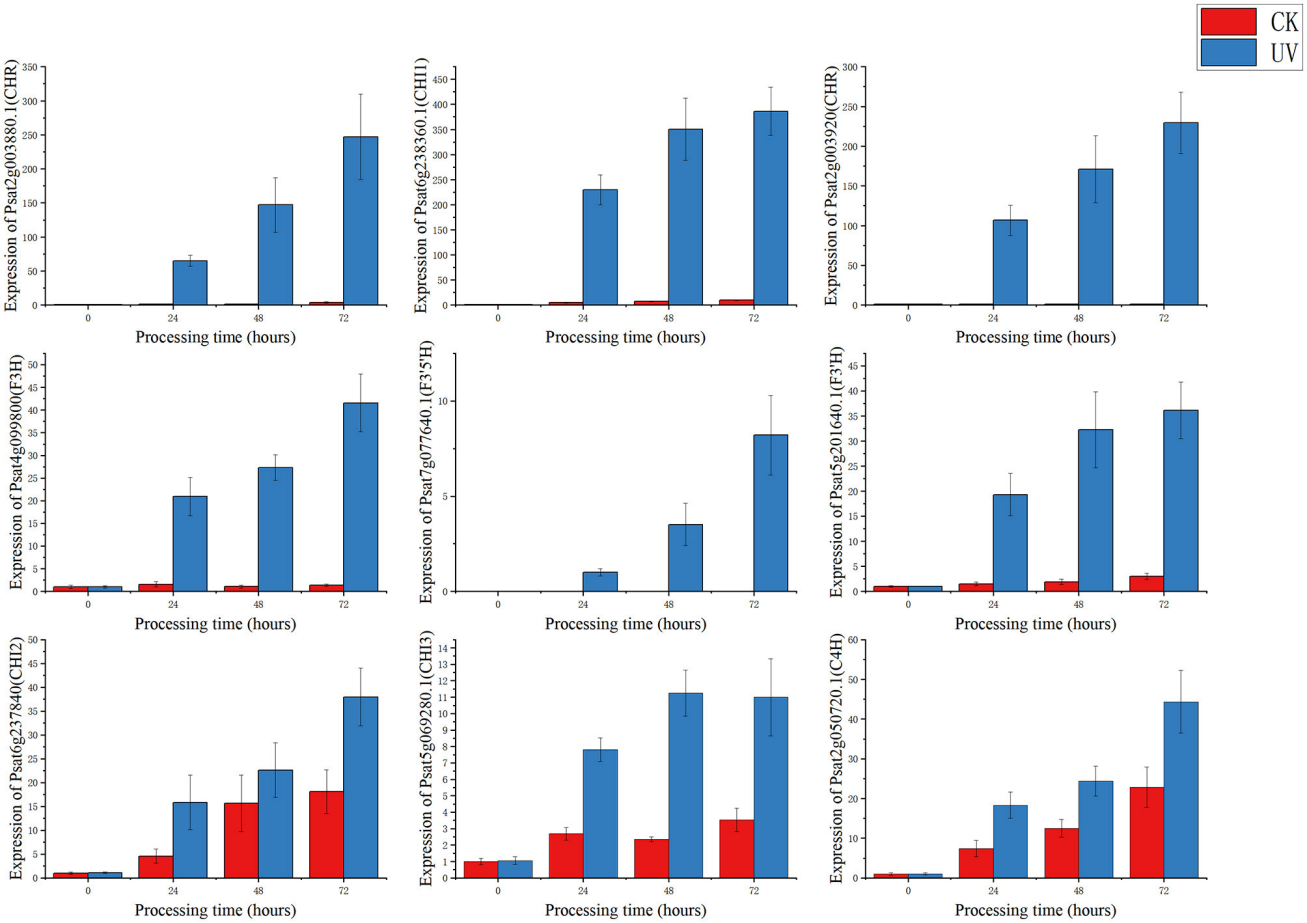
RT-qPCR regulating the gene expression status of differential proteins.

**Figure 7:**
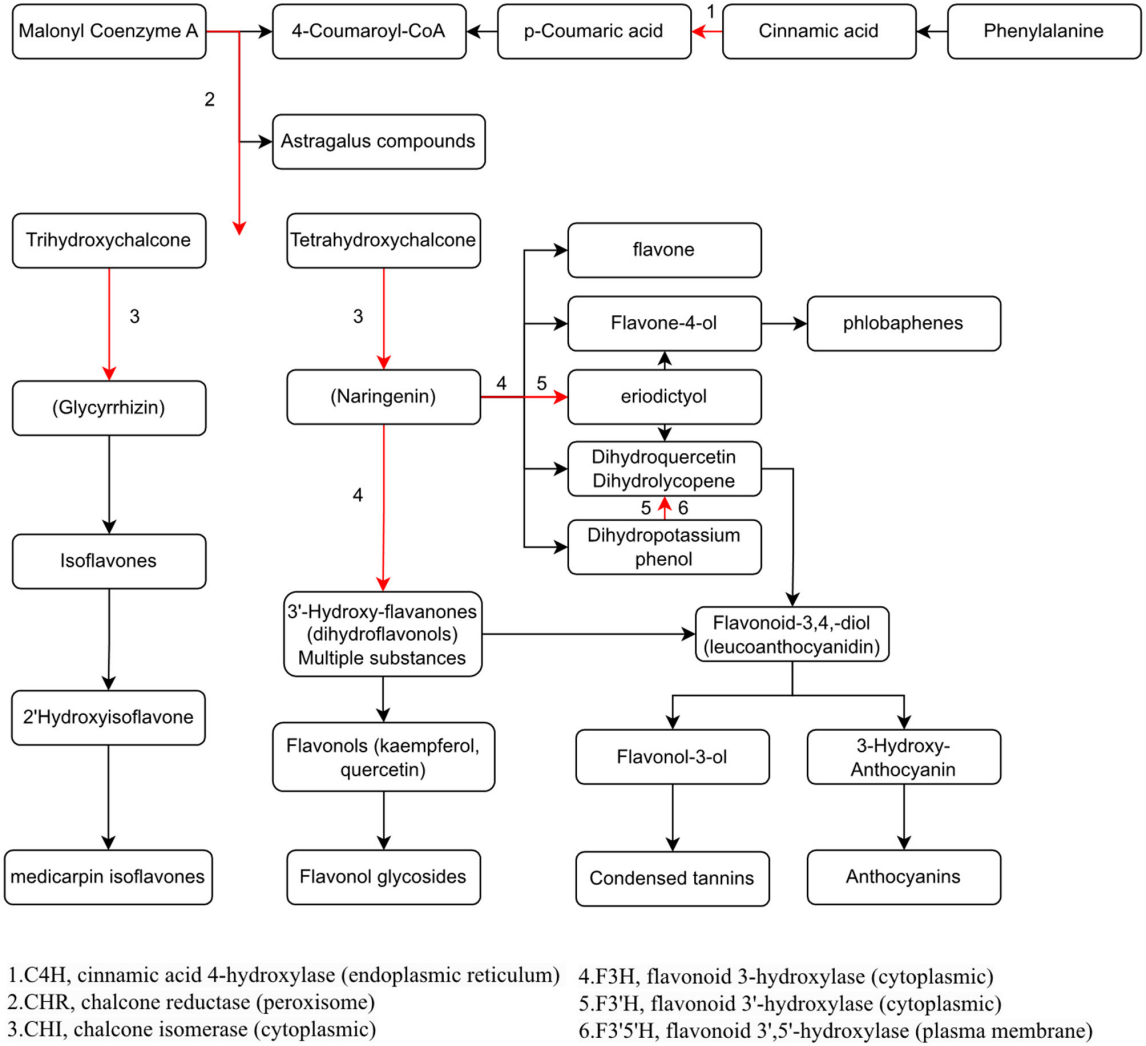
Visualization of the effect of UV-B on the metabolic pathway of flavonoids in pea seedlings.

To study the relationship between transcript and protein levels in pea seedlings after UV-B irradiation, key enzymes (differential proteins) involved in flavonoid metabolism were selected and their regulatory genes were subjected to RT-qPCR to determine changes in their transcript levels (Figure 6). UV irradiation increased the flavonoid content of plants, and this change occurred immediately after irradiation. This was confirmed by our study (Figure 6), where the expression of genes regulating these differential proteins exhibited a substantial increase, and most of the increase was the greatest at 24 h after UV-B treatment. And these differentially expressed genes and proteins involved in the metabolic pathway of flavonoids are given in Figure 7, which summarized the metabolic process of flavonoids in the plant body and indicated the location of the differential proteins. UV-B irradiation could cause substantial and overall increase in the content of flavonoids in pea seedlings, and the location and mechanism of its action were assessed.

The expression of genes regulating CHR in the UV group (Psat2g003880.1 and Psat2g003920) was 56.4- and 34.6-fold higher than that in the CK group; the corresponding protein contents were 14.6- and 3.5-fold higher than those in the CK group, respectively. The expression of genes regulating C4H (Psat2g050720.1) in the UV group was 1.9-fold higher than that in the CK group, and the corresponding protein content was 1.4-fold higher than that in the CK group. C4H and CHR were located in the essential pathway of flavonoid synthesis. Cinnamic acid is p-hydroxylated by C4H to form p-coumaric acid (53), and an increase in CHR level enhances anthocyanin synthesis (54). C4H is the key to the phenylpropanoid pathway, which is the most important point that influences the increase of flavonoid compounds. The effect of UV-B on flavonoids is also reflected in C4H, and the increase of C4H can also promote the accumulation of flavonoids. The expression of genes regulating CHI1 (Psat6g238360.1), CHI2 (Psat6g237840), and CHI3 in the UV group (Psat5g069280.1) was 4.81, 2.1, and 3.1 times higher than that in the CK group, respectively. The corresponding protein contents were 3.3, 1.2, and 1.6 times higher than those in the CK group, respectively. CHI is located in the main pathway of flavonoid synthesis and acts simultaneously in several branching pathways. In our study, UV-B irradiation simultaneously activated CHI1, CHI2, and CHI3, which are all critical in the synthesis of flavonoid substances, affecting the synthesis of naringenin and glycyrrhizin, as shown in Figure 7, where naringenin can be fully regulated by CHI, while glycyrrhizin is only regulated by CHI1 and CHI3 regulation (55), and an increase in CHI in the pathway would eventually advance the synthesis of medicarpin isoflavones and flavonol glycosides. The expression of genes regulating F3H (Psat4g099800) was 4.65-fold higher in the UV group than in the CK group. The expression of genes regulating F3'H (Psat5g201640.1) in the UV group was 12.1-fold higher than that in the CK group; the corresponding protein content was 1.7-fold higher than that in the CK group. The expression of genes regulating F3'5'H (Psat7g077640.1) in the UV group was increased by 8.21-fold, and its expression was not detected in the CK group. F3H, F3'H, and F3'5'H are a series of enzymes with synergistic effects, all of which play an important role in the accumulation of anthocyanins (56, 57), and together they are enhanced under the influence of UV-B, promoting the formation of anthocyanins, flavonol glycosides and other substances. In addition, upstream genes CHI and F3H are genes of early unbranched fragments of the flavonoid synthesis pathway, and their expression levels can also induce the expression of downstream genes (58), indirectly helping to activate the flavonoid synthesis process. The transcription and translation levels of biological genes are often not synchronized but differ (59), and this was confirmed in the present study. In the present study, the magnitude of variation at the transcriptional level of individual genes was much greater than that at the translational level, and the differences could even reach 10's of folds, probably because transcriptional and translational mechanisms do not coincide in the organism (60).

## 4. Conclusions

In this study, we treated pea seedlings with UV-B for 10 min daily and investigated its effects in terms of both physiological and biochemical indicators and proteomics. UV-B irradiation significantly reduced the weight and length of edible parts of growing pea seedlings (*p* < 0.05), decreasing them by 5.7 and 14.2%, respectively; however, ascorbic acid content, flavonoid content, and root length significantly increased by 19.0, 112.4, and 11.4% (*p* < 0.05), respectively. Further proteomic analysis revealed that UV-B irradiation increased the levels of MIOX and GME in the cytoplasm and decreased the levels of extracellular GME in the ascorbic acid metabolic pathway. During phytohormone signaling, UV-B irradiation affected the levels of transporters and response proteins, where the content of AUX1 in Golgi apparatus was significantly reduced and the content of SAUR72 in mitochondria was substantially increased (*p* < 0.05). UV-B irradiation significantly increased the levels of key enzymes in the biosynthetic pathway of flavonoid compounds in pea seedlings and acted mainly upstream. This study demonstrated that UV-B irradiation significantly increased (*p* < 0.05) the levels of key enzymes CHI, F3'5'H, and F3H located in the cytoplasm; F3'H in the plasma membrane; C4H in the endoplasmic reticulum; and CHR in the peroxisome. In further studies in the future, the focus will be on the study of the effect of gene overexpression or RNA silencing state of the protein in question on the plant, thus exploring the mechanism of its effect on the plant.

## Data availability statement

The datasets mass spectrometry proteomics data for this study can be found in the ProteomeXchange Consortium via the PRIDE partner repository with the dataset identifier PXD038486. https://www.ebi.ac.uk/pride/archive/projects/PXD038486.

## Author contributions

XF conducted the investigation, validation, and edited the manuscript. YX conducted the experiments, data compilation, did the visualization and analysis, and wrote the first draft of the manuscript. ML conducted project management and provided methodology and funding. All authors contributed to manuscript revision, read, and approved the submitted version.
